# QUALICOPC, a multi-country study evaluating quality, costs and equity in primary care

**DOI:** 10.1186/1471-2296-12-115

**Published:** 2011-10-20

**Authors:** Willemijn LA Schäfer, Wienke GW Boerma, Dionne S Kringos, Jan De Maeseneer, Stefan Greß, Stephanie Heinemann, Danica Rotar-Pavlic, Chiara Seghieri, Igor Švab, Michael J Van den Berg, Milena Vainieri, Gert P Westert, Sara Willems, Peter P Groenewegen

**Affiliations:** 1 NIVEL, Netherlands Institute for Health Services Research, Utrecht, The Netherlands; 2 Department of Family Medicine and Primary Health Care, Ghent University, Ghent, Belgium; 3 Hochschule Fulda - University of Applied Sciences, Fulda, Germany; 4 ULMF- University of Ljubljana, Ljubljana, Slovenia; 5 Laboratorio MeS, Istituto di Management, Scuola Superiore Sant'Anna, Italy; 6 RIVM- National Institute for Public Health and the environment, Bilthoven, The Netherlands; 7 Tranzo, Tilburg University, Tilburg, The Netherlands; 8 Radboud University Nijmegen Medical Centre, Nijmegen, The Netherlands; 9 Department of Sociology and Department of Human Geography, Utrecht University, Utrecht, The Netherlands

## Abstract

**Background:**

The QUALICOPC (Quality and Costs of Primary Care in Europe) study aims to evaluate the performance of primary care systems in Europe in terms of quality, equity and costs. The study will provide an answer to the question what strong primary care systems entail and which effects primary care systems have on the performance of health care systems. QUALICOPC is funded by the European Commission under the "Seventh Framework Programme". In this article the background and design of the QUALICOPC study is described.

**Methods/design:**

QUALICOPC started in 2010 and will run until 2013. Data will be collected in 31 European countries (27 EU countries, Iceland, Norway, Switzerland and Turkey) and in Australia, Israel and New Zealand. This study uses a three level approach of data collection: the system, practice and patient. Surveys will be held among general practitioners (GPs) and their patients, providing evidence at the process and outcome level of primary care. These surveys aim to gain insight in the professional behaviour of GPs and the expectations and actions of their patients. An important aspect of this study is that each patient's questionnaire can be linked to their own GP's questionnaire. To gather data at the structure or national level, the study will use existing data sources such as the System of Health Accounts and the Primary Health Care Activity Monitor Europe (PHAMEU) database. Analyses of the data will be performed using multilevel models.

**Discussion:**

By its design, in which different data sources are combined for comprehensive analyses, QUALICOPC will advance the state of the art in primary care research and contribute to the discussion on the merit of strengthening primary care systems and to evidence based health policy development.

## Background

Recently, the World Health Organisation (WHO) for the European Region developed 'Health 2020', a new policy oriented vision. It addresses recent challenges to health, such as non-communicable diseases and negative consequences of the ageing of the population, with a specific focus on health inequalities [1] The EC funded study QUALICOPC (Quality and Costs of Primary Care in Europe) fits well within 'Health 2020', as it aims to evaluate primary care (PC) in Europe in terms of quality, equity and costs of care. The primary level of health care systems has the potential to effectively address the core elements of 'Health 2020', namely social determinants of health and non-communicable diseases. PC can be defined as generalist care being the first level of access to the professional health care system. PC is characterised by its accessibility for the population, irrespective of the nature of health problems, and is provided near patients' homes. Besides providing curative care, PC also offers preventive care and health education. In many European countries, general practitioners (GPs) or family physicians are the main providers of PC. Furthermore, PC includes a variety of providers such as general internists, general paediatricians and gynaecologists. Besides, also dentists, pharmacists, therapists (e.g. physiotherapists and speech therapists), and mental health care workers (e.g. community psychiatrists and psychologists) provide PC [2,3].

Results of the study will inform decision makers about PC systems that have a better quality and cost balance than others and thus enable them to better manage healthcare reforms [4]. Until now, evidence on the benefits of PC is inconclusive and insufficiently takes the diversity and complexity of European health care systems into account [5]. This article explains the background and design of the QUALICOPC project.

A major step in the global attention for PC has been the WHO Declaration of Alma Ata from 1978. The Declaration stressed the importance of creating and sustaining a strong primary (health) care (PHC) system, not just as a part of the health care system, but in particular linked to other sectors as well [6]. The impact of the PHC concept in the industrialised countries has been limited. In Greece PC was reorganized on the basis of these principles [7]. Also, after a revolution changing the regimes, Spain and Portugal used PC principles to develop PC systems with family physicians [7]. 'Alma Ata' has inspired countries in Europe to develop their own structure of the 'first line' health care services. After the collapse of the Communist regimes in 1991, countries in Central and Eastern Europe were forced to fundamentally restructure their health care systems, including PC [8,9]. Today, strengthening PC is worldwide probably higher on the agenda than ever [10]. It is expected to be an effective response to effects of the current economic crisis on health and health care [11].

The policy strategy towards PC reinforcement is often based on the notion that a strong PC system benefits a nation's health and health care system. PC has the potential to contribute to overall health system performance and health [5].

### What is known about benefits of PC?

Previous studies have found better performance among health care systems based on solid PC systems [12-24]. Scientific research, both international comparisons and within the United States, has shown that well developed PC systems have better coordination and continuity of care and better opportunities to control costs [2,12,21,25-27].

A recent review on the relationship between PC and health outcomes and costs reports that in PC oriented countries the population experiences better outcomes and lower costs are incurred [28]. A variety of studies have demonstrated that the supply of primary health care doctors and the ongoing relationships between patients and their GPs are associated with total costs of care. This was true for the adult population as well as among elderly in the USA [29-32].

Furthermore, research from the USA has shown that availability of GPs and Family Physicians and first contact care are associated with reduced unnecessary care (avoidable hospitalisation) and increased accessibility [32-35]. Avoidable hospital admissions can be used as an indicator of health care performance. An admission is avoidable when a relatively expensive hospital admission for a certain condition could have been prevented by effective and/or accessible primary health care. The availability of GPs and insurance coverage for PC are related to lower rates of avoidable hospitalisations [36].

Also, regions with a higher PC doctor density have a healthier population and reduce the negative effects of social inequality [37]. The evidence of a relationship with the structure and strength of PC at national level and equity is however scarce. Equity is usually studied by analysing large national health interview surveys. A study of OECD countries [38] could not substantiate a relationship with PC. Concerning the effects of strong PC on equity results are inconclusive. Until now, no such effects have been clearly demonstrated in international studies [38-40]. However, there are indications that access to care for minority groups is better in well developed PC systems [41].

A negative effect is that patient satisfaction seems to be lower in health care systems with regulated access to specialist services by gate keeping [42,43].

Several studies, predominantly from the USA, have shown positive effects of PC on health outcomes [5,14]. Health policies aimed at strengthening PC are associated with better levels of health [14]. Strong PC is associated with better health outcomes such as lower rates of all-cause, heart disease, and cancer mortalities [14,44].

In the early 1990's an EU funded project studied the profiles of general practice in Europe. Considerable variation was found in the task profile of PC providers in health care systems in European countries. There were contrasts between regions within Europe and GPs within countries showed large differences in their service profiles [45-48]. The international differences were related to characteristics of the health care systems, such as the GPs' employment status, gate keeping role and mode of remuneration [45,46,49].

In summary, previous studies have found relationships between PC and different health care system outcomes. However, from the European perspective, the currently available evidence on the effects of PC should be considered with care due to the limited generalisability of the results to the European context. These studies have usually included only a selection of EU countries and, additionally, covered non-European OECD countries. Furthermore, so far, little is known about the mechanisms that link aggregate structural elements of health care systems with performance of health care systems. This would demand a deeper insight in professional behaviour of health care workers and the expectations and actions of patients.

More in-depth analyses are needed to substantiate abovementioned findings. Better international comparative data and analyses of good practices will produce information to policy makers and those responsible for provision of services about the drivers of strong PC [50-52].

### Objectives

Since the Declaration of Alma Ata, many European countries share the goal of initiating or sustaining a strong PC system as part of their health care system. As a result there is a demand for benchmark information and a growing tendency to learn from foreign experiences. Based on these notions, the QUALICOPC (Quality and Costs of Primary Care in Europe) project has been designed. The project receives co-funding of the European Commission under the "Seventh Framework Programme".

The QUALICOPC project aims to evaluate PC systems in Europe against criteria of quality, equity and costs. QUALICOPC looks at what a strong PC system entails and aims to provide an answer to the question:

#### What effect does the strength of a primary care system have on the performance of health care systems?

To this end, the organisation of PC at GP practice level and national structures for PC will be related to overall health care system goals, to indicators of the process quality of PC service provision, and to indicators of the quality of PC as perceived by the users of services. The strength of a PC system is determined by the degree of development of a combination of PC functions both at structure level (governance, economic conditions and workforce development) and at process level (access, continuity of care, coordination of care and comprehensiveness of care) in the context of its health care system [5,40,53].

Since 'quality' is a broad concept, its use in the context of PC deserves explanation. Firstly, quality can be related to the structure of care (referring to characteristics such as equipment and human resources), the process of care (the actual delivery of care) or the outcomes (consequences of the process in terms of e.g. health status or patients' evaluations) of it. This division is based on Donabedian's well-known framework.

Secondly, generic and specific dimensions of PC quality should be distinguished [54]. Generic dimensions are those applicable to all health care services; examples are equity, accessibility and user friendliness of services and effectiveness (both clinical effectiveness and interpersonal effectiveness). Specific dimensions are typically applicable to PC systems:

- continuity of care (longitudinal care; episodic continuity)

- coordination and integration (with other professionals and levels of care)

- scope of services (broad range of curative and preventive services)

- community orientation

To make the insights of this study tangible, good practices in PC organisation will be identified and disseminated. The insights and lessons produced by the study, in addition to the policy consequences that will be explored, will help decision makers to shape PC systems optimally, given the possibilities, needs and restrictions.

## Methods/design

### Overall design and hypotheses

**Figure 1:**
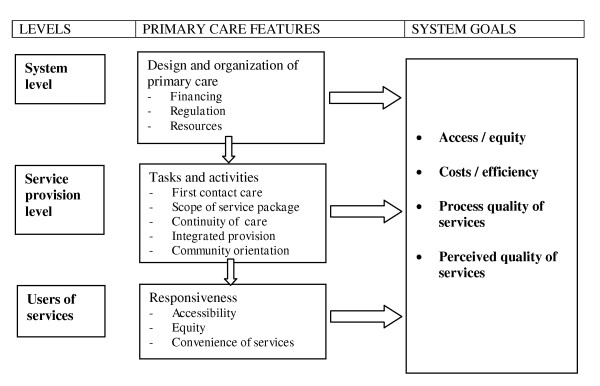
**Elements of the study and their inter-relations**.

In order to fully understand the underlying mechanisms of PC leading to health system outcomes, this study distinguishes three levels of care. The first level is the system level of PC, encompassing features such as financing, governance and resources. The second level is the provision level, characterised as the delivery of care process at GP practice level. GPs can be seen as the core providers of PC. The third level, are the users of PC services. The features at these three different levels are expected to, directly and indirectly, contribute to health, access/equity, costs, process quality of services and perceived quality of services. The (inter)relations between the different levels and their features and the outcomes are visualised in Figure 1.

A number of hypotheses will be tested in this study, concentrating on different domains: quality of service provision, patients' perceived quality of care, costs, equity, avoidable hospitalisation and good practices. The main hypotheses that will be tested are:

1. The degree of organisation of PC practices (e.g. higher skill mix and better organisation of out-of-hours care) is positively associated with the process quality of their services; (system → service provision)

2. A strong PC orientation at structure level is positively associated with the degree of organisation of practices and the process quality of services; (system → service provision)

3. Process quality of PC services is positively associated with patient evaluations of PC quality; (service provision → perceived quality of services)

4. The degree of the organisation of PC practices in combination with quality of the PC process is negatively associated with the incidence of avoidable hospitalisations; (system & service provision → process quality)

5. The strength of PC systems (in terms of strong PC orientation at structural level, good organisation of PC practices and high quality of PC services) is negatively associated with total health care expenditures; (system & service provision → costs/efficiency)

6. A strong PC orientation at structure level is positively associated with access at the practice level and patient perceived equality in access by socio-economic status; (system & service provision & access→ equity)

7. The scale of PC organisational units is associated with lower costs and higher quality. (service provision → costs & process quality & perceived quality)

8. Process innovation (case and disease management, patient-centered care, integration of prevention) is associated with lower costs and higher quality; (service provision → costs & process quality & perceived quality)

9. Delegation and substitution of tasks within PC is associated with lower cost and higher quality. (service provision→ costs & process quality & perceived quality)

### Data collection

**Figure 2:**
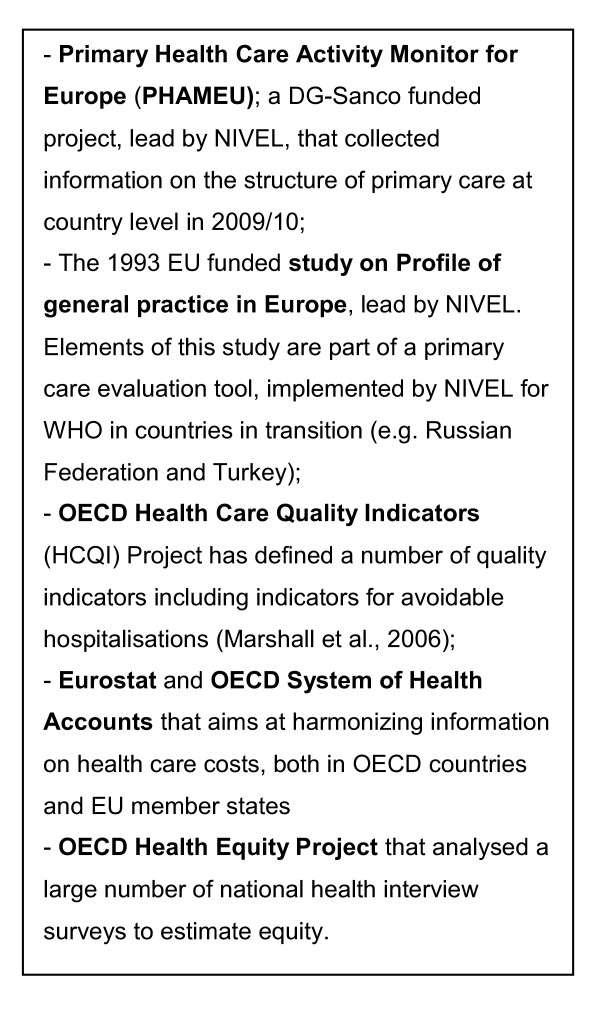
**Existing data sources to be used in QUALICOPC**.

Multiple methods of data collection are used in this study. First, the study builds upon existing knowledge, by making use of international studies which have previously invested in collecting evidence on PC at the system (structure) level (see Figure 2).

Secondly, new data are collected, using a cross-sectional survey methodology in a multi-actor design. The multi-actor design makes it possible to directly connect information on PC practices to information provided by patients of these practices. The survey consists of:

- A survey among GPs as core providers of PC collecting data at the process level. The survey also collects information on involvement and relations with other PC providers; part of the survey will be modelled on essential elements of the 1993 study mentioned in Figure 2[47].

- A survey among patients that were treated at these PC practices to gather data on the process and outcome level; the methodology for this design has been developed and tested in the context of earlier WHO projects (e.g. performed in Turkey and Russia) [55,56]. The survey among patients consists of two questionnaires: one about patients' experiences and one about patients' values. Measuring what patients find important enables the weighing of their experiences [57].

### Setting and sampling

Data is collected in 31 European countries (including all 27 EU Member States: Austria, Belgium, Bulgaria, Cyprus, Czech Republic, Denmark, Estonia, Finland, France, Germany, Greece, Hungary, Ireland, Italy, Latvia, Lithuania, Luxembourg, Malta, Netherlands, Poland, Portugal, Romania, Slovakia, Slovenia, Spain, Sweden, UK and two Candidate Member States: Turkey and Iceland and Norway and Switzerland) and in three non-European countries (Australia, Israel and New Zealand). In each country we aim to realize a response of 220 GPs. In Cyprus, Iceland, Luxembourg and Malta the desired response is lower (around 75). In each country we aim to draw a nationally representative sample of GPs. Initially, this means that a simple random sampling procedure is used, drawing a random sample from the national register of GPs (if available). To avoid the inclusion of multiple GPs which are subject to the same practice variables, only one GP per practice will be included. In countries where a national register is not available a multistage sampling procedure is used e.g. by combining registers from different regions or municipalities. Furthermore, in large countries with differences in health care systems across regions, we selected a number of nationally representative regions and subsequently randomly selected GPs within these regions.

The patient survey will include patients above the age of 18 visiting a GP who filled in the questionnaire. Hence, in this study there is a focus on patients in PC who actually visited the practice. This means that the outcomes of the survey will represent the views of users of PC, rather than the general population. The questionnaires for patients will be distributed through the PC physicians who participate in the GP survey. The 220 physicians will be asked if a fieldworker may visit the practice to distribute questionnaires to patients who have consulted them. In practice, on a set date the fieldworker will visit the practice and ask patients to fill in the questionnaire in the waiting room, until a response of 10 patients has been reached. Per country we aim for a response of 1800 patients for the experiences questionnaire and 220 for the values questionnaire (see section 'Questionnaire development' for explanation on the questionnaires). In Cyprus, Iceland, Luxembourg and Malta the desired response is respectively 720 and 80 patients. In some countries where additional funds are available, a larger response will be realised to make comparison between different regions possible. In each practice the fieldworker will ask the first 9 patients, who are willing to participate, to fill in the experiences questionnaire and the 10^th ^patient to fill in the values questionnaire.

Based upon earlier research it is known that the total numbers per country are sufficient to relate country characteristics, PC practice variables and patient evaluations; moreover numbers are large enough to produce reliable country level estimates of patient evaluations of PC [58]. Several measures will be taken to acquire sufficient response to the survey. Firstly, per country, a national expert will be commissioned as a national coordinator of the fieldwork. Secondly, the coordinator will be asked to organise acquisition of national support from professional organisations for the study. Thirdly, financial resources will be made available to serve as incentives for GPs. Finally, in each country we will have extra versions of the patients' questionnaires available in the languages of the largest ethnic minority groups.

**Figure 3:**
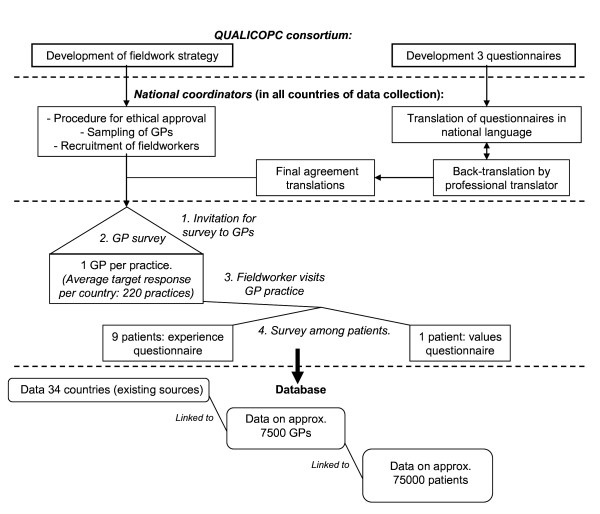
**Overview of the QUALICOPC study protocol**.

An overview of the methodologies used in the QUALICOPC study is shown in Figure 3.

### Questionnaire development

To collect new data required for this study, questionnaires are already developed. The questionnaires need to contribute to the provision of usable data for a variety of topics on PC in Europe. For the GP questionnaire these topics concern activities and tasks of PC providers, process quality of PC and accessibility of PC at the organisational level. The patient questionnaire is aimed at gaining insight into the evaluation of services from the point of view of patients/clients by measuring the perceived quality of care, perceived access to care and actual cost barriers to PC.

To come to well-founded questionnaires several steps were taken. First, a framework, including important aspects regarding the process and outcomes of care, was defined. For the GP questionnaire the framework of Kringos et al (2010) was used. For the patient questionnaire a framework was used based upon the Consumer Quality Index of GP care [59]. Secondly, a search in scientific databases and on the Internet on existing questionnaires on the topics included in the frameworks was performed. Thirdly, the questions from the selected questionnaires were grouped according to the topics of the identified frameworks. Fourthly, gaps were identified by experts on the different research topics (such as equity and costs). It was evaluated for which topics appropriate questions were lacking. For these topics, new questions were formulated. Based upon the findings from the third and fourth step, three questionnaires were developed: one for GPs and two for patients, distinguishing patients' experiences and patients' values. The questionnaires were then piloted in three countries (Belgium, Slovenia and the Netherlands). Based on the findings adjustments were made and consensus on the final questionnaires was reached based on experts' opinions (see section 'pilot'). Specifications on the outcomes of the search strategy, questionnaire development and questionnaires will be published separately.

The survey among GPs includes self-reported involvement in curative and preventive tasks and questions on the type and organisation of the practice, integrated provision of services and aspects of workload and use of time. The patients experience survey contains questions about the patients' backgrounds, distance to the PC practice, choice of doctor, copayments for services, time for the patients, availability of health education, experiences with services of the practice or centre, experiences with their own doctor and aspects of care coordination. The patients value questionnaire contains questions about the patients' backgrounds and their values regarding GP care.

As the survey will be held among GPs and patients in 31 European and 3 other countries, the questionnaires will be translated from the English master version into the national languages. Also, to reach the largest groups of ethnic minorities within the countries, some extra versions in languages such as Arabic will be made available for patients. An independent 'forth and back translation' procedure will be used.

### Pilot

A pilot was held to test the process of completing the survey in the GP practice and to test the relevance and comprehensibility of both questionnaires. Questions regarding the process that were addressed are e.g.: How long does it take to fill in the questionnaires? Are GPs and patients easily willing to participate? By testing comprehensibility of the questionnaires we tried to answer questions like: Are the instructions on the questionnaire understood by all respondents? For the closed questions, are all reasonable alternatives included for the respondents? The pilot was held in three countries in Europe (Belgium, the Netherlands and Slovenia) among a small sample of GPs and patients. GPs and patients were surveyed in the GP practice setting.

### Data handling

All questionnaire data will be centrally processed in the Netherlands. The questionnaires will have a uniform design and a closed answering format to allow optical reading technology for data entry. The data will be analysed initially to construct new variables to be used in the analyses for the hypotheses. This guarantees unity in the way important variables, such as process quality of PC and patient evaluations of PC quality, are defined.

### Data analysis

The data collected in this study will be integrated by using statistical models for hierarchically structured data in multilevel models [60,61]. Multilevel models enable to partition the variation in (e.g.) patient perceived quality of care into three parts:

- a part related to the individual patients (related to personal background, health status etc.)

- a part related to the PC practices they visit (e.g. related to the range of services that these practices provide and the process quality of the practices)

- a part related to the health care system of the countries (e.g. related to structural aspects of PC and the strength of PC).

Apart from studying general patterns and trends we will identify good practices which in a statistical sense are the outliers in the statistical analysis. Analyses will be made on the combinations of traits and circumstances that explain their position.

Data analysis techniques will include: data reduction by scale construction using factor analysis, reliability analysis and ecometrics; single level regression and correlation to analyse the relations at country level between PC structure and quality, cost and equity variables; multilevel analysis to relate country, practice and patient levels.

The use of multilevel statistical analysis (MLA) is essential in this study, in particular where survey data (from GPs and patients) are integrated with aggregate data at health care system level. The MLA approach has specifically been developed for these situations, where units on which variables are measured are nested within larger (higher level) units; such as patients within GP practices, or GPs within a country's health care system. MLA allows analysing variables at the country level and at the GP (practice) level at the same time.

### Personal data confidentiality

For the survey among GPs random samples will be drawn of GPs from available lists or registers. Patients will be approached in the practices. Procedures which apply for this use of registers in each country and for survey research among patients will be identified and carefully observed. When necessary, we will apply for ethical approval in the participating countries. The survey is anonymous; respondents (physicians and patients) do not need to fill in their name. To be able to link the data of GPs to the countries and their patients, we will use identification numbers. In no publication results will be reported that can be related in any way to individuals or locations.

### Investigators

The research team consists of experienced researchers, with backgrounds in health services research, economic analysis, patient evaluation surveys, survey design, statistical modelling and PC research. The team has extensive experience in research on international comparisons of health care systems. Each of the involved research institutes are leading on one of the main study topics (equity, costs or efficiency, process quality of services, perceived quality of services) which fits within their expertise.

## Discussion

The evidence on the effects of strong PC systems is inconclusive. Reforms favouring PC systems are based on the plausibility of effects rather than on its base of evidence. The available evidence is from studies with a limited focus, and not representing the diverse situations of health care in the countries of Europe. The QUALICOPC project will considerably contribute to this base of evidence and thus advance the state of the art of (primary) health services research.

The outcomes of the QUALICOPC project will be used to inform the European Union and other international organisations, such as the WHO, but particularly also national governments. The deeper insights, provided by this project, in specific elements of PC organisation and provision which have a positive effect on performance of health systems in general, will contribute to more effective health policy.

QUALICOPC uses an ambitious methodology integrating different levels of care by the use of existing databases and surveys among GPs and their patients. Using elements from the 1993 Task Profile study will not just provide information on changes that have occurred since then, the innovative element is adding the patient's perspective, thus increasing the chances of meaningful interpretations.

QUALICOPC will use a survey methodology in a multi-actor design, allowing to connect the information on PC practices with information provided by patients from these practices and system level information. The use of these state-of-the-art methods is expected to serve as a 'model of good practice' for future health services studies.

### QUALICOPC Worldwide

Based on the network of the QUALICOPC consortium, several research institutes from countries with a PC system comparable to European countries were invited to participate in the QUALICOPC study. Three non-European countries have raised funding and will participate in this study: Australia, Israel and New Zealand. For the study it will improve the evidence base for the mechanisms of PC systems and their effect on health care system performance measures. In addition, a broader international participation will provide the study a deeper insight in the national strategies of PC systems, professional behaviour of health care workers and the expectations and actions of patients around the world.

## Conclusion

Demographical changes, technological developments and rising expectations bring about many challenges for European health systems in the coming decades [62]. In Europe, countries are looking for solutions to create more coherence and coordination in care to address the problem of a lack in responsiveness to the needs of populations. PC is seen as the part of the health care system where this problem can be tackled to a large extent [10]

The variety of models of organisation and provision of health care services found in Europe, are favourable circumstances to undertake sound and comprehensive studies on the merits of PC for health care systems in general. The rich diversity of the structure and financing of European health systems, makes this setting a laboratory for comparative research and a pool of good practices [63]. The QUALICOPC study benefits of this situation by thorough analysis of PC at three levels in 31 European countries. The impact of QUALICOPC is boosted as a result of its strategy to combine previous work (which itself had already a good impact) with new elements, one of which being the measurement of the way PC affects equity in health care. With the applied study design, this project will be able to answer the question "What effect does the strength of a primary care system have on the performance of health care systems?"

## Competing interests

The authors declare that they have no competing interests.

## Authors' contributions

WLAS, WGWB, DSK and PPG wrote the manuscript. JDM, SG, SH, DRP, CS, IS, MVDB, MV, GW, SW reviewed the draft manuscript. All authors read and approved the final manuscript.

## Pre-publication history

The pre-publication history for this paper can be accessed here:

http://www.biomedcentral.com/1471-2296/12/115/prepub
